# Temporal pattern of *Fos* and *Jun* families expression after mitogenic stimulation with FGF-2 in rat neural stem cells and fibroblasts

**DOI:** 10.1590/1414-431X2023e12546

**Published:** 2023-09-08

**Authors:** A.C. Mosini, P.C. Mazzonetto, M.L. Calió, C. Pompeu, F.H. Massinhani, T.K.E. Nakamura, J.M. Pires, C.S. Silva, M.A. Porcionatto, L.E. Mello

**Affiliations:** 1 Laboratório de Neurobiologia, Departamento de Fisiologia, Escola Paulista de Medicina, Universidade Federal de São Paulo, São Paulo, SP, Brasil; 2 Laboratório de Neurobiologia, Departamento de Bioquímica, Escola Paulista de Medicina, Universidade Federal de São Paulo, São Paulo, SP, Brasil; 3 Instituto D’Or de Pesquisa e Ensino, São Paulo, SP, Brasil

**Keywords:** Neural stem cells, Fibroblasts, Immediate early genes, FGF-2

## Abstract

Intense stimulation of most living cells triggers the activation of immediate early genes, such as *Fos* and *Jun* families. These genes are important in cellular and biochemical processes, such as mitosis and cell death. The present study focused on determining the temporal expression pattern of *Fos* and *Jun* families in fibroblasts and neural stem cells of cerebellum, hippocampus, and subventricular zone (SVZ) of rats of different ages at 0, 0.5, 1, 3, and 6 h after stimulation with fibroblast growth factor (FGF)-2. In neonates, a similar expression pattern was observed in all cells analyzed, with lower expression in basal condition, peak expression at 0.5 h after stimulation, returning to baseline values between 1 and 3 h after stimulation. On the other hand, cells from adult animals only showed *Fra1* and *JunD* expression after stimulation. In fibroblasts and hippocampus, *Fra1* reached peak expression at 0.5 h after stimulation, while in the SVZ, peak level was observed at 6 h after stimulation. *JunD* in fibroblasts presented two peak expressions, at 0.5 and 6 h after stimulation. Between these periods, the expression observed was at a basal level. Nevertheless, *JunD* expression in SVZ and hippocampus was low and without significant changes after stimulation. Differences in mRNA expression in neonate and adult animals characterize the significant differences in neurogenesis and cell response to stimulation at different stages of development. Characterizing these differences might be important for the development of cell cultures, replacement therapy, and the understanding of the physiological response profile of different cell types.

## Introduction

It is well known that neurogenesis in neonate and adult rodents differs in several manners such as in the processes of proliferation and differentiation, the duration of which is extended in adult cells (1). Such particularities of neonate and adult neurogenesis are directly influenced by gene expression. During development and learning processes, neuronal activation triggers the expression of immediate early genes (IEGs) responsible for coding transcription factors such as members of the Jun and Fos families (2).

The Fos and Jun protein families can work individually or through numerous combinations of dimers to generate the transcription factor activating protein-1 (AP-1) complex. The transcription factor AP-1, which activates the transcription of a range of numerous and diverse genes, is related to cell differentiation and proliferation, learning, motor control, and cognition (3,4). It is known that Fos and Jun expression is regulated by the activation of mitogen-activated protein kinase (MAPK) and phosphorylation of cAMP response element-binding protein (CREB) (5,6). Several growth factors, including fibroblast growth factor (FGF), activate MAPK signaling pathway.

Numerous studies using mice and rats show Fos and Jun gene expression in several regions of the central nervous system when animals are exposed to a wide range of stimuli like trauma (7), water stress (8), fear (9), odors, intraparenchymal injections of various compounds (10,11), and visual stimuli (12). Basal expression of *c-Fos* has been studied in several species, including mice, rats, cats, monkeys, and humans. Although *c-Fos* basal levels are relatively low due to its mRNA instability, transcription occurs rapidly (5 min) and continues for up to 15-20 min (13). IEGs have been used as markers of neural activity during memory formation. However, the lack of a temporal comparison between them hinders the interpretation of some results (14). In addition, little is known regarding the comparative expression of these genes in neural stem cells after mitogenic stimulation in different cell lines and at different developmental stages. For this purpose, we chose fibroblasts as the standard, likely one of the best characterized cells in culture, and neural stem cells, due to their relevance to replacement therapy as well as their intrinsic proliferative restriction in adults.

Knowing that FGF-2 is a critical factor for both neural stem cells and fibroblasts, we decided to compare the temporal expression patterns of Fos and Jun gene families in these cell types at different developmental stages to understand some of the changes that are essential for memory formation. Despite the remaining proliferative capacity of both fibroblasts and some neural cells, we hypothesized that there are marked differences in gene expression responses when comparing these two different age groups.

## Material and Methods

### Animals

To determine the temporal pattern of gene expression of *Fos* and *Jun* families in neural stem cells and fibroblasts from neonates and adult rodents, we assessed their expression after *in vitro* mitogenic stimulus with FGF-2. For that, we used 70 neonate (6-day-old; P6) and 130 adult (8-week-old) rats (Wistar). The protocols used here were approved by the UNIFESP Ethics Committee on the Use and Care of Animals (CEP No. 5165260716). Neonate rats were euthanized with scissors and adults were anesthetized with ketamine (60 mg/kg) and xylazine (15 mg/kg) intraperitoneally followed by decapitation with a guillotine.

### Cell isolation and culture

#### Fibroblasts

The primary cell culture of fibroblasts was established using dermal fragments from the abdominal region of neonate and adult rats. Dermal fragments were stored in a plastic tube with DMEM/F-12 (GibcoBRL, USA). Inside the laminar flow, the dermal fragments from adults and neonates were placed in Petri dishes with a 5 mL of 0.5% trypsin (Gibco) solution in PBS (1×) for 30 min at room temperature. After the incubation time, the fragments were dissociated with scissors and incubated again in an enzymatic solution (5 mL of HEPES/RPMI (Gibco) supplemented with 1 mM sodium pyruvate, 0.1 mg/mL DNAse I (Thermo Fisher, USA), 2.75 mg/mL collagenase (Gibco), and 1.25 mg/mL hyaluronidase (Sigma Aldrich Corp., USA) for 3 h at room temperature. The samples were then dissociated with a glass pipette, passed through a 40-μm mesh filter (Corning, USA), centrifuged for 10 min at 272 *g* at room temperature, and the pellet was resuspended in 10 mL of supplemented medium containing DMEM/F-12 1:1 (Gibco), 10% of fetal bovine serum (FBS; Cultilab, Brazil), 1% penicillin (Gibco), and 1% streptomycin (Gibco). The cells were then incubated in 75-cm^2^ bottles in 5% CO^2^ at 37°C. After 24 h of plating, the entire culture medium was withdrawn, and 10 mL of fresh medium was added. Every four days the culture medium was changed.

#### Cerebellar neural stem cells

The cerebellum from P6 rats (Wistar) was dissected and dissociated by incubation with trypsin 1× (Gibco) for 5 min at 37°C. The cell suspension was centrifuged for 3 min at 272 *g* at room temperature, the supernatant was discarded, and the pellet was resuspended in 1 mL of supplemented medium containing DMEM/F12 1:1 (Gibco), 2% B27 supplement (Gibco), 20 ng/mL EGF (Sigma), 20 ng/mL FGF2 (RandD Systems, USA), 1% penicillin/streptomycin (Gibco), and 1% glutamine (Sigma). Finally, cells were plated in 75-cm^2^ bottles (TPP, Switzerland) in 5% CO_2_ and at 37°C. Every three days, the culture medium was changed.

#### Neural stem cells of subventricular zone and hippocampus

Neural stem cells were obtained from 8-month-old rats (Wistar). Briefly, rats were euthanized, and their brains were removed, the subventricular zone (SVZ) and the hippocampus were dissected, and the cells were dissociated by incubation with trypsin 1× (Gibco) for 5 min at 37°C. After enzymatic and mechanical dissociation, cells were strained in a 40-μm mesh filter (Corning) and plated in flasks coated with Poly-HEMA (Sigma) in supplemented medium containing DMEM/F12 1:1 (Gibco), 2% B27 supplement (Gibco), 20 ng/mL EGF (Sigma), 20 ng/mL FGF2 (RandD Systems), 1% penicillin/streptomycin (Gibco), 1% glutamine (Sigma), and 5 µg/mL heparin (Sigma) in 5% CO_2_ and at 37°C. Every three days the culture medium was changed.

### Stimulus with FGF-2

The established period for cell growth was 15 days for neonatal cells and 45 days for adult cells. After this period, the culture medium was completely changed to a supplemented culture medium without EGF and FGF-2 for 24 h. After this period, cells were stimulated by adding 20 ng/mL of FGF-2 to the medium for different time intervals: 0 h (without stimulus); 0.5, 1, 3, and 6 h.

### RNA extraction, reverse transcription, and quantitative PCR

A total RNA sample from all samples was extracted by Trizol^®^ Reagent (Invitrogen, USA) according to the manufacturer's instructions. The RNA concentration was measured on the NanoDrop ND-2000 Spectrophotometer (NanoDrop Technologies, USA), and RNA integrity was verified by electrophoresis in 1% agarose gel. Reverse transcription was performed using 2 µg total RNA and before the reaction, samples were treated with RQ1 RNAse-Free DNAse (Promega, USA) to eliminate genomic DNA following the manufacturer's protocol. cDNA synthesis was performed using ImProm-IITM Reverse Transcriptase, ImProm-IITM Reaction Buffer, Oligo (dT)15 Primer (0.5 mM), dNTP mix (10 µM), Recombinant RNasin^®^ Ribonuclease Inhibitor, MgCl_2_ (25 mM) (Promega), and RNA-free water to complete 20 µL. The thermal conditions used for amplification in a thermocycler (Eppendorf, Germany) were: 25°C for 5 min, 42°C for 60 min, and 70°C for 15 min. qPCR was performed using Fast SYBR^®^ Green Master Mix (Thermo Fisher) and 7500 Fast Real-Time PCR System (Applied Biosystems, USA). The reaction was carried out in 10 µL final volume using a mixture of 50 ng/µL of cDNA, 5 µL Fast SYBR^®^ Green Master Mix, Forward Primer (10 mM), and Reverse Primer (10 mM). The thermal cycling used for qPCR was 95°C for 10 min, 40× 95°C for 15 s, 60°C for 1 min, and 72°C for 30 s. The dissociation curve was done at 95°C for 1 min, 60°C for 30 s, and 95°C for 30 s. Relative expression levels were calculated using the 2^-ΔΔCt^ method and normalized using reference genes that show more stability in geNorm analysis.

### Statistical analysis

Data were analyzed using GraphPad Prism^®^ software version 5.0.3 (USA). Comparisons were made with ANOVA followed by the Tukey test. The established levels of statistical significance were P<0.05 and P<0.001.

## Results

### Expression pattern of neonatal cells

#### Neonatal fibroblasts

**Figure 1:**
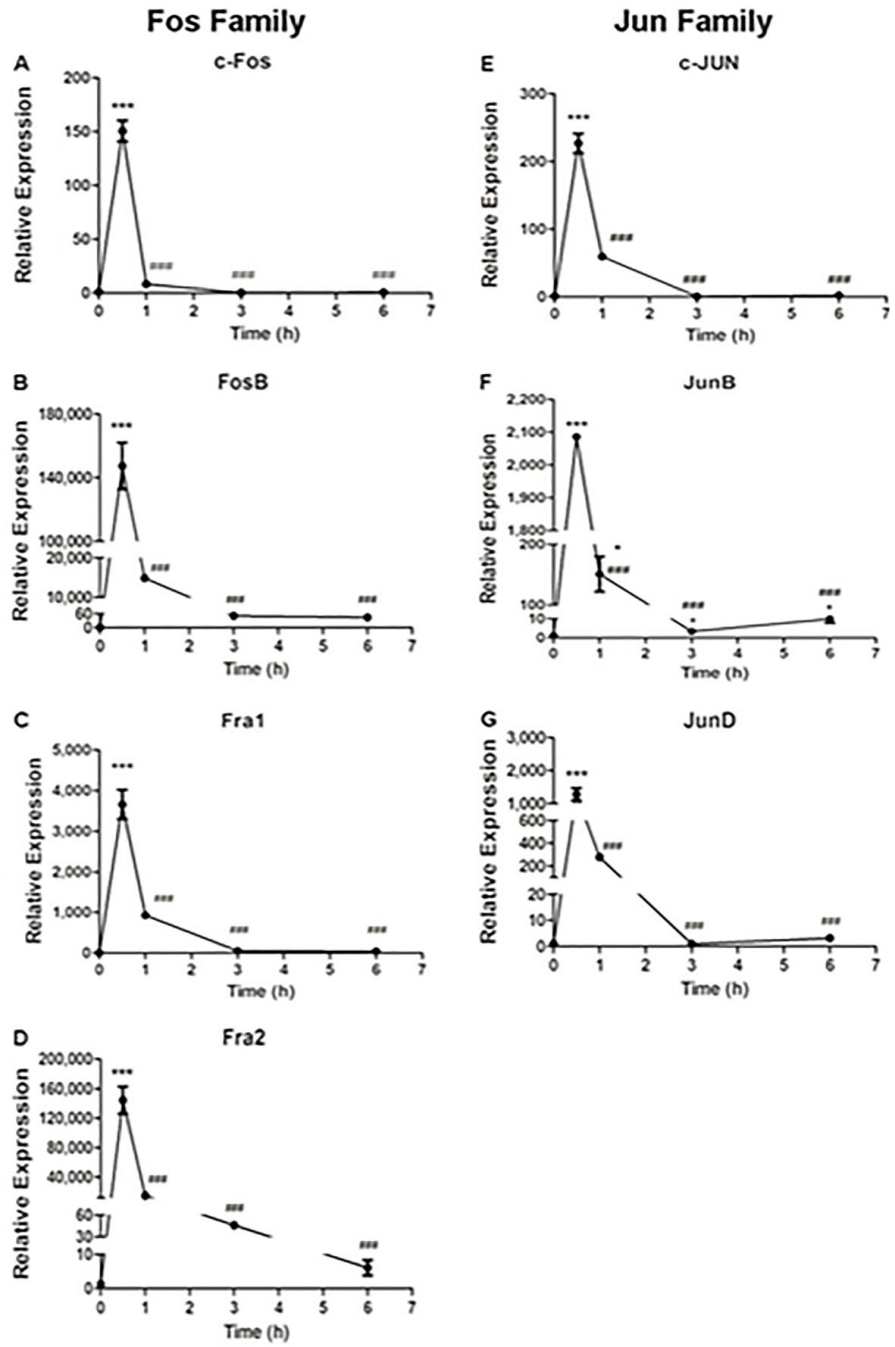
Relative expression profile of the *Fos* and *Jun* family genes in fibroblasts from neonates after fibroblast growth factor (FGF)-2 stimulation. Data are reported as mean and SD. *P<0.05; ***P<0.001 compared to baseline condition; ^###^P<0.001 compared to 0.5 h time point; ^+^P<0.05 compared to time 1 h (ANOVA with Tukey post-test).

Neonatal dermal fibroblasts had upregulated expression of all genes from the *Fos* and *Jun* families, peaking at 0.5 h after stimulation with FGF-2 (Figure 1), followed by a decrease at 1 h. For *FosB* and *Fra2* (Figure 1B and D), the peak expression at 0.5 h was 150,000 times greater than at baseline. Likewise, *JunB* and *JunD* (Figure 1F and G) showed an increase in expression of 2,200 and 1,200 times, respectively, that of basal at the same time point. The expression of *Fra1* (Figure 1C) increased about 3,500-fold, while *c-Fos* and *c-Jun* (Figure 1A and E) showed 150 and 220-fold greater expressions at 0.5 h compared to basal expression levels. Expression of all genes analyzed returned to baseline levels 3 h after the stimulus. All genes except *JunD* presented significant differences in expression at 1, 3, and 6 h compared to time 0.5 h. For JunD, there was a significant difference only at 1 h compared with the expression at 0.5 h after the stimulus.

#### Neonatal cerebellar neural stem cells

**Figure 2:**
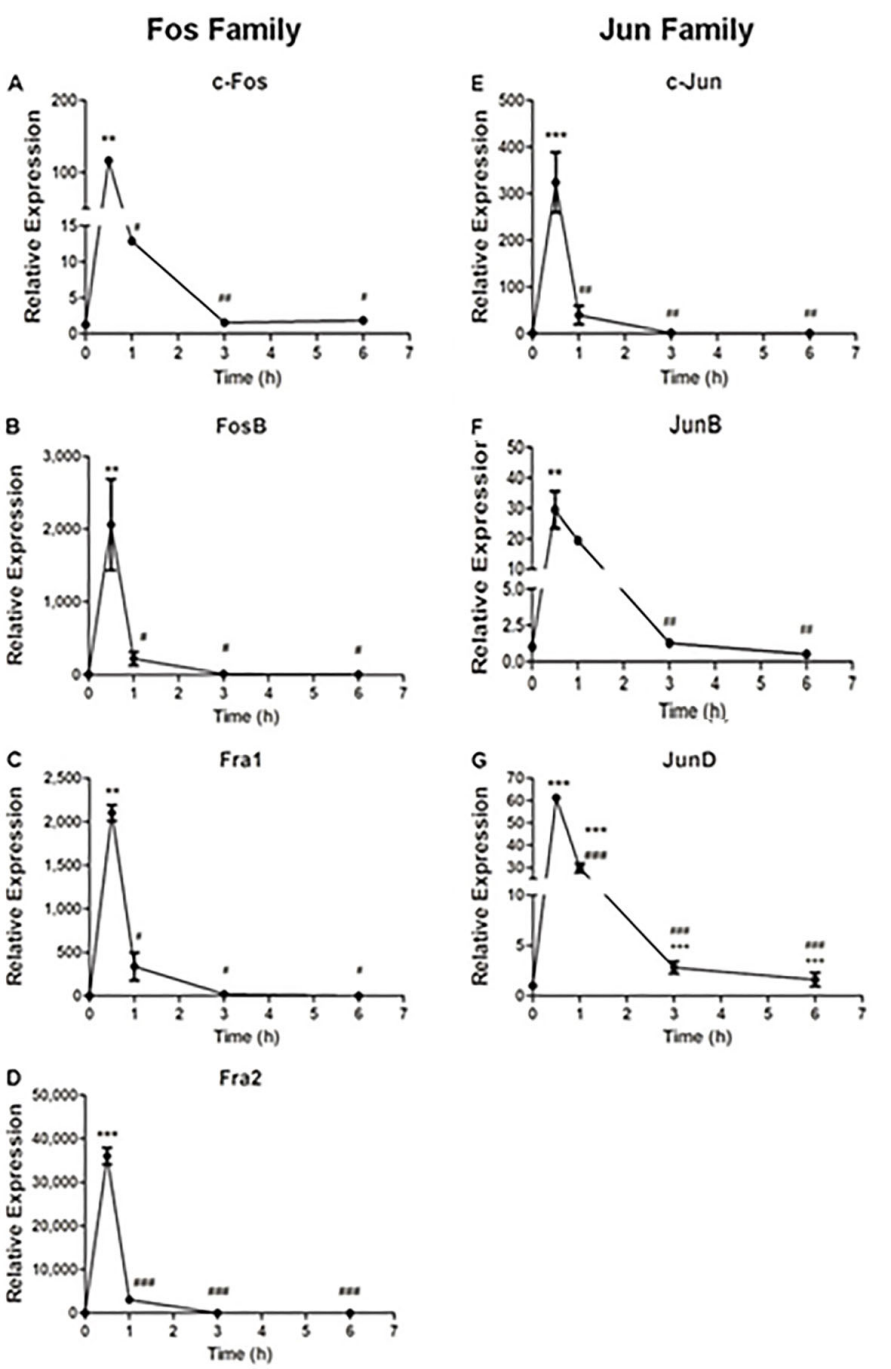
Relative expression profile of the *Fos* and *Jun* family genes in cerebellar stem cells of neonates after fibroblast growth factor (FGF)-2 stimulation. Data are reported as mean and SD. **P<0.01; ***P<0.001 compared to baseline condition; ^#^P<0.05; ^##^P<0.01; ^###^P<0.001 compared to time 0.5 h; ^+++^P<0.001 compared to time 1 h (ANOVA test with Tukey post-test).

Similar to fibroblasts, the gene expression of the *Fos* and *Jun* families in neural stem cells extracted from the neonatal rodent cerebellum increased and peaked at 0.5 h (Figure 2) after mitogenic stimulation with FGF-2. The expression returned to baseline values at 1 h (Figure 2B-E), except for the *c-Fos, JunB,* and *JunD* genes (Figure 2A and F-G), which returned to baseline only at 3 h after the stimulus. Regarding the magnitude of expression at 0.5 h, *Fra2* (Figure 2D) was the gene that presented the greatest increase in expression in this cell type, reaching 3,500-fold of the basal condition. Likewise, the expression of *FosB* and *Fra1* (Figure 2B and C) was 2,000 times greater at the same time point. For *c-Fos* and *c-Jun* (Figure 2A-E), the increase was respectively 120-350 times greater than the basal condition. Finally, *JunB* and *JunD* (Figure 2F and G) were the genes that presented the smallest increases, albeit significant (respectively 30 and 60 times), in expression at 0.5 h. For all genes, there was a significant difference between time points 1, 3, and 6 h in relation to time point 0.5 h, except for *JunB*, where the difference in relation to time point 0.5 h only existed for time points 3 and 6 h. In contrast, *JunD* was the only gene that had a significant difference in expression between time point 1 h compared with time points 3 and 6 h.

### Expression pattern of adult cells

Fibroblasts and neural stem cells from hippocampus and SVZ of adult animals showed a pattern of expression markedly different from that of neonate animals. Surprisingly, there was no expression of several genes of the *Fos* (*c-Fos, FosB*, and *Fra2*) and Jun (*c-Jun* and *JunB*) families. The only genes for which we found differences in the expression pattern were *Fra1* and *JunD*. The expression pattern of those genes, however, varied extensively between the different cell types, and a standard common expression profile was not present.

**Figure 3:**
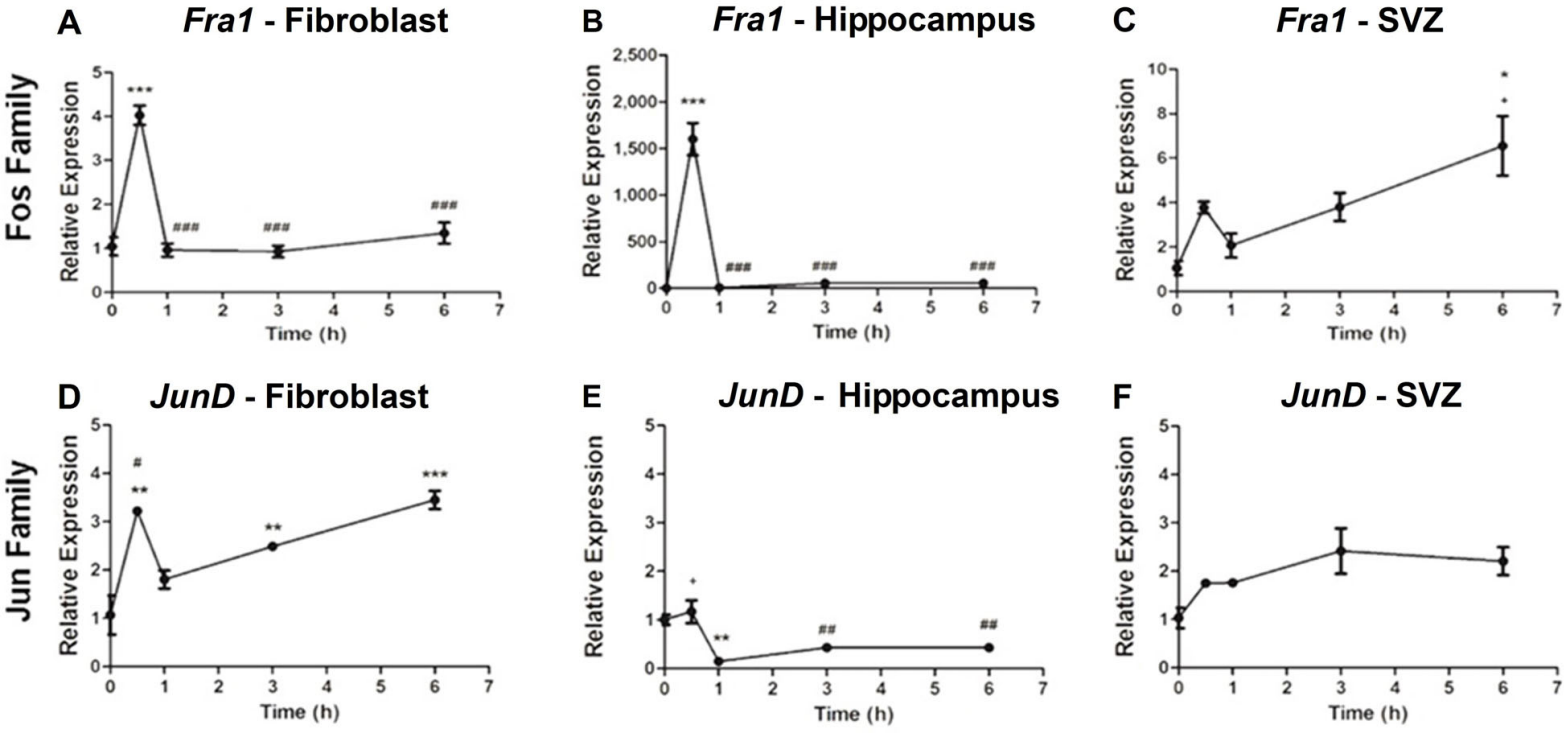
Relative expression profile of the *Fos* and *Jun* family genes in neural stem cells and fibroblasts of adult rats after fibroblast growth factor (FGF)-2 stimulation. Data are reported as mean and SD. *P<0.05; **P<0.01; ***P<0.001 compared to baseline condition; ^#^P<0.05; ^##^P<0.01; ^###^P<0.001 compared to time 0.5 h; ^+^P<0.05 compared to time 1 h (ANOVA test with Tukey post-test).

*Fra1* in both fibroblasts and hippocampus neural stem cells (Figure 3A and B) showed a peak expression at 0.5 h following FGF-2 stimulation. In fibroblasts, this increase in expression was 4 times higher than basal expression, while in the hippocampus this increase reached 1,600 times. In both cell types, expression of *Fra1* returned to baseline levels at 1 h. For neural stem cells isolated from the SVZ, expression of *Fra1* reached statistical significance only 6 h after stimulation (Figure 3C), which was also the peak expression for this gene in this cell type (by 6-fold).

For *JunD* in both fibroblasts and hippocampus stem cells (Figure 3D and E), peak expression was also at 0.5 h after stimulation. For the subsequent time points, however, there was a markedly different gene expression profile in these two cell types. In fibroblasts, there was a significant subsequent downregulation of gene expression followed by further increases (by 3.5-fold) up to 6 h after stimulation when expression was at a similar level to that seen at 0.5 h (Figure 3D). In adult neural stem cells from the hippocampus, there was a significant decrease in *JunD* expression at 1, 3, and 6 h after stimulation compared both to basal and 0.5 h levels (Figure 3E). *JunD* expression in the SVZ did not achieve statistical significance at any of the time points.

## Discussion

The *Fos* gene family is involved in many physiological functions, such as cell cycle control and proliferation, while the *Jun* gene family is related to differentiation and cell death. When combined, they form the AP-1 complex (4). Fibroblasts and cerebellar neural stem cells from neonates had a similar pattern of gene expression. We observed an increase in mRNA expression for all investigated genes of the *Fos* and *Jun* families in neonatal cells after 0.5 h of mitogenic stimulus induced by FGF-2, returning to basal levels after 1 h. As for fibroblasts and neural stem cells from SVZ and hippocampus from adult animals, however, our results showed an expression of only two genes, *Fra1* and *JunD*.

It is known that neonate and adult neurogenesis differ in many aspects, including the proliferation and differentiation processes (15). These particularities are directly influenced by gene expression. Although we did not quantify cell proliferation in the cultures, we speculated that the reported upregulation of gene expression may contribute to the intense neurogenesis and cell proliferation of neonates.

Adepoju et al. (16) observed that FGF-2 activated the expression of c-Fos and c-Jun protein at 1 h post-stimulation, whereas *c-Fos* levels peaked between 1 and 3 h and declined markedly at 6 h after stimulation and *c-Jun* expression was sustained for more than 12 h. In contrast, we observed that neural stem cells derived from cerebellum of neonate rats expressed all genes of the *Fos* and *Jun* families after 0.5 h of mitogenic stimulus, returning to basal levels after 1 h. These differences can be due to the type of material analyzed, the species, and the neurodevelopmental phase.

Kang et al. (17) analyzed the *c-Fos* expression (gene and protein) in response to FGF-2 in the human embryonic stem cell line MizhES1. About 1 h after stimulation, *c-Fos* gene mRNA was clearly induced and expression of c-Fos protein reached its highest expression levels at 2 h after stimulation. These results for *c-Fos* mRNA corroborate with our data that showed an increase of *c-Fos* after 0.5 h of mitogenic stimulus in neonatal fibroblasts and neural stem cells.

In contrast to the extensive evidence of *c-Fos* expression at 0.5 h after stimulation in adult rats (for a review, see 18), we did not observe *c-Fos* expression at this period in neural stem cell culture from adult animals. According to Salehi et al. (19), the inducibility of *c-Fos* declines with increasing age, supporting the notion that *c-Fos* expression widely reflects the cellular responsiveness at the transcriptional level.

Several studies indicate that *Fos* and *Jun* mRNA expression may in fact be above baseline levels already after 20 min of stimulation of the visual cortex of rats subjected to various periods of light exposure after 24 h of dark rearing (20). *c-Fos* transcriptional activation occurs within a few minutes of growth factor stimulation (13) and, according to Sheng and Greenberg (21), *c-Fos* transcription is undetectable at 0.5 h after growth factor treatment (such as FGF-2) in adult neuronal cells, corroborating our study.

Rosenfeldt et al. (22) showed that the level of *c-Fos* mRNA increased dramatically in fibroblasts from human foreskin specimens and peaked 50 to 60 min after contraction was initiated in stressed collagen matrices. Additionally, Deguchi et al. (23) showed that the level of *c-Fos* mRNA expression reached a maximum at about 0.5 to 1 h after oxygen reperfusion and declined to basal levels after 2 h in cultured human fibroblasts. Similarly, our data from neonatal fibroblasts showed a rapid *Fos* and *Jun* expression, peaking at 0.5 h after FGF-2 stimulation and returning to basal levels after 1 h. In further agreement with others, Oshima et al. (24) showed that *c-Fos* mRNA was readily inducible in young cells at 1 h after stimulation with serum, while this response was ablated in senescent cultures.

In addition, many articles show *c-Fos* expression in astrocytes. In an ischemic model in primary culture of cortical astrocytes, an increased expression in the range of 0.5-2 h (peak at 1 h) is observed. Bradykinin, forskolin, and IL-1 exposure also promotes an increase in *c-Fos* expression after 1 h in cell culture (2).

Our results showed that *JunD* expression in fibroblasts and the SVZ was sustained and at a stable level in adult animals. Expression in the hippocampus was lower but similarly stable. *FosB*, *Fra1, c-Jun,* and *JunB* were not expressed at any time after stimulation in any of these cells. Our findings corroborate those of Kallunki et al. (25), who demonstrated that *JunD* is expressed in neurons and glial cells and displays a basal expression in most cells in the nervous system. Kovary and Bravo (26) demonstrated in Swiss 3T3 cells that there is a variation in the AP-1 complex composition, in which initial dimers are formed by *c-Fos/JunB* and later on, by *Fra1/c-Jun* and *Fra2/JunD*. Therefore, we believe that the *Fra1/JunD* response encountered here in adult cells might also be associated to a similar specificity of response. Among other processes, the AP-1 complex is involved in cell cycle regulation. For a review on this topic, see (27).

The restricted expression of only *Fra1* and *JunD* in our cell cultures derived from adult animals might also be related to the expression of FGF receptors in these cells. Indeed, it has been reported that there is a progressive diminution in the expression of FGF receptors in various regions of the central nervous system of adults (28,29).

Another relevant aspect to be discussed is the difference in gene expression between neonate and adult animals regarding the increase in expression between baseline and 0.5 h (Table 1). In both fibroblasts and neural stem cells from neonate animals, all genes of the *Fos* and *Jun* families presented a significant increase in expression at 0.5 h after stimulation with FGF-2. Despite differences in expression values, *Fra1, Fra2, c-Jun*, and *JunD* showed a similar decrease in expression in the order of 4-5-fold at 1 h after stimulation (compared to 0.5 h). However, for *c-Fos*, *FosB*, and *JunB*, the decrease was 10-15-fold, emphasizing the peak expression at 0.5 h, while the Jun family genes decrease ranged between 0-8-fold at the same time point. We speculate that this difference in the intensity of gene expression over time might be related with the functions associated with those genes.

**Table 1:** Increase in gene expression in all the regions studied after 0.5 h of the stimulus in relation to basal condition.

Genes	Neonates		Adults		
	Fibroblasts	Cerebellum	Subventricular zone	Hippocampus	Fibroblasts
*Fos* famil*y*					
*c-Fos*	150×	120×	-	-	-
*FosB*	150,000×	2,000×	-	-	-
*Fra1*	3,500×	2,000×	3×	1,600×	4×
*Fra2*	150,00×	3,500×	-	-	-
*Jun* family					
*c-Jun*	220×	350×	-	-	-
*JunB*	2,200×	30×	-	-	-
*JunD*	1,200×	60×	2×	-	3×

The dash indicates genes that did not express.

Here, we showed that expression of the *Fos* and *Jun* gene families might change according to age of the animal and type of cell and tissue, indicating that neurogenesis of neonates and adults has many fundamental differences at the molecular level, and we speculate that this may even influence behavioral and cognitive outcomes.
